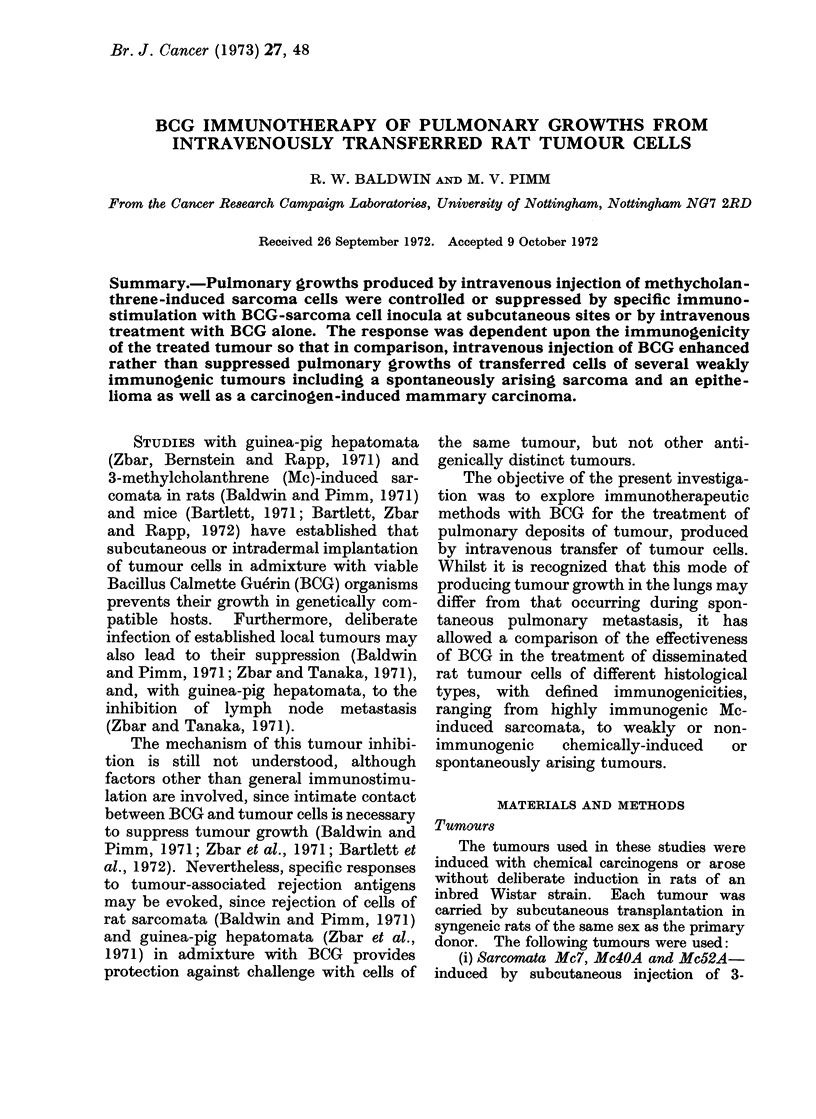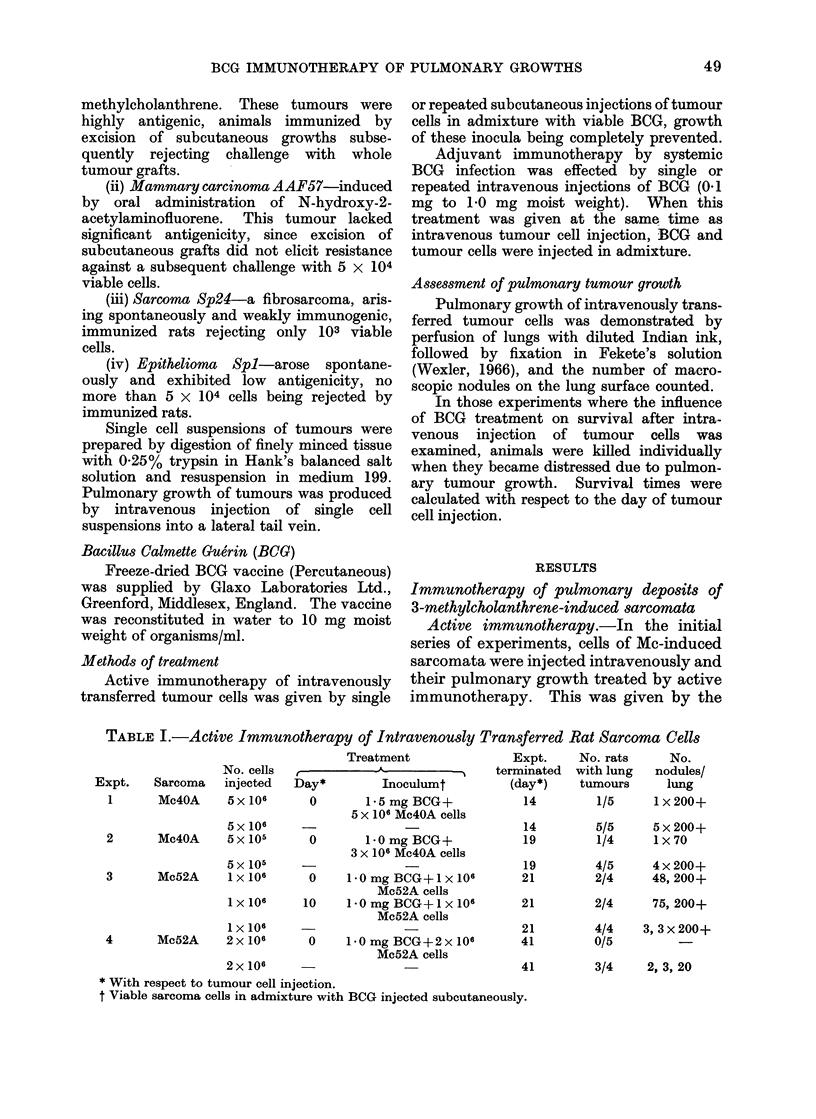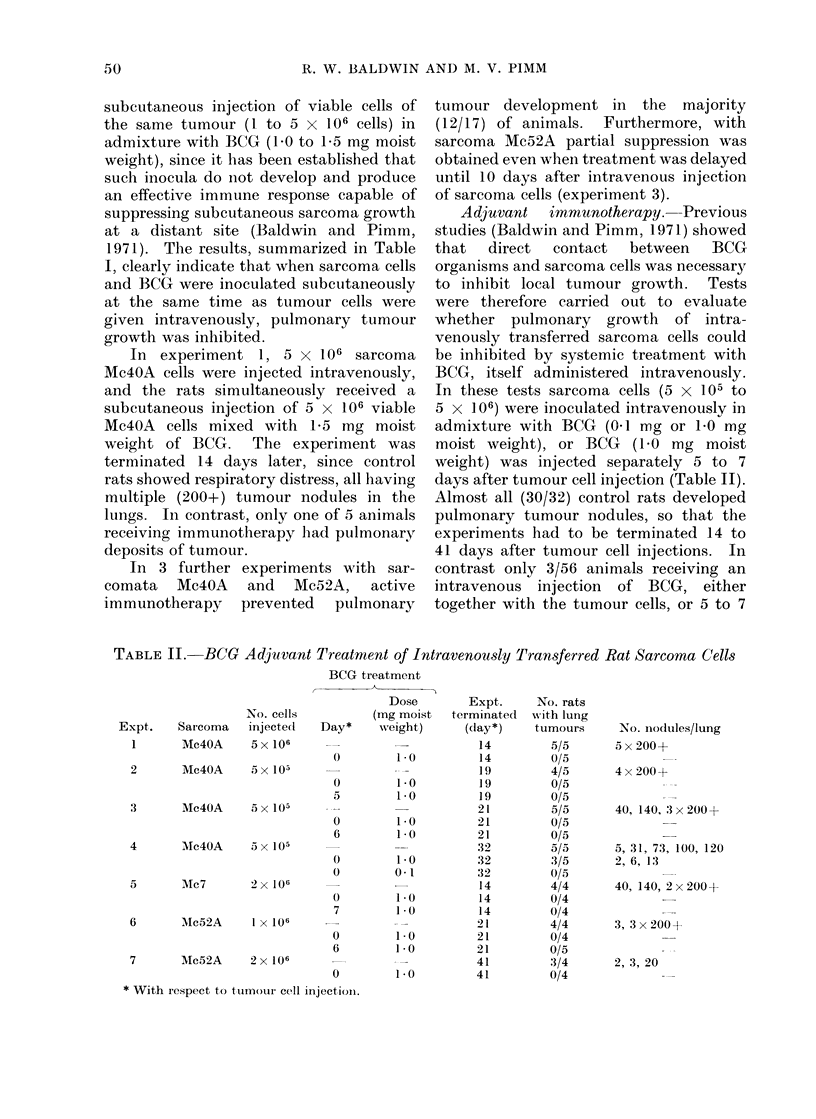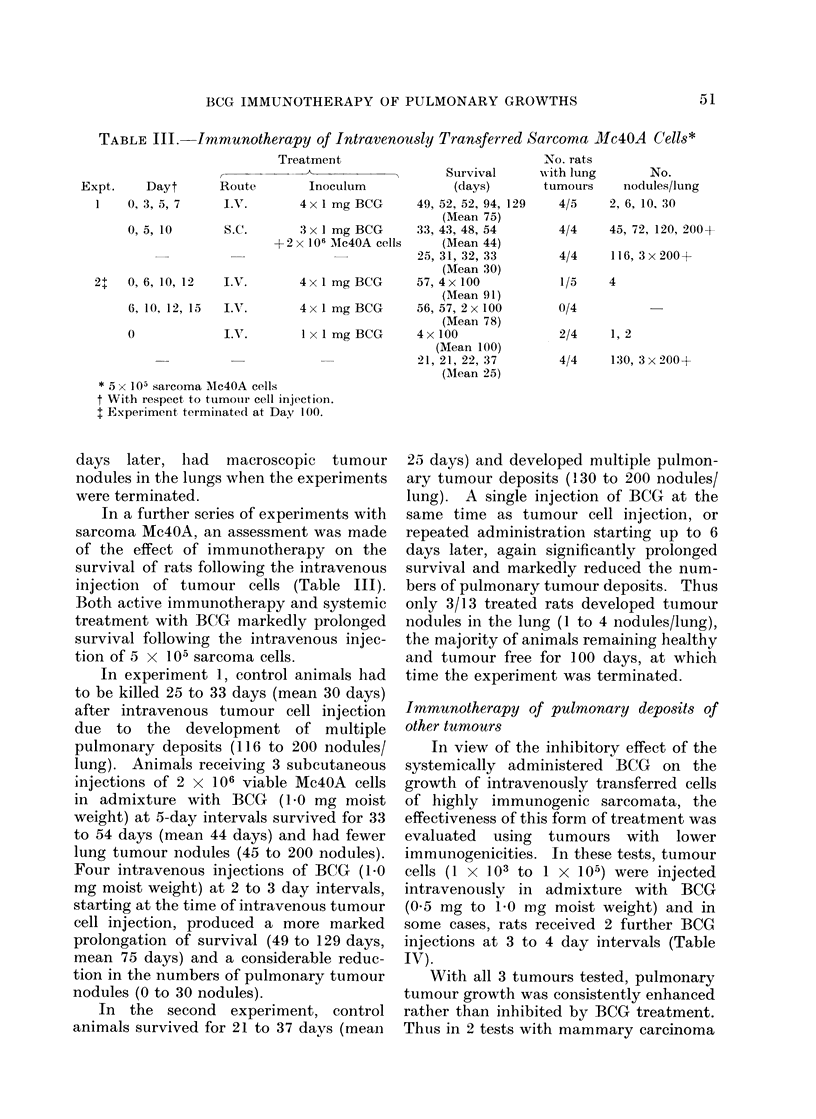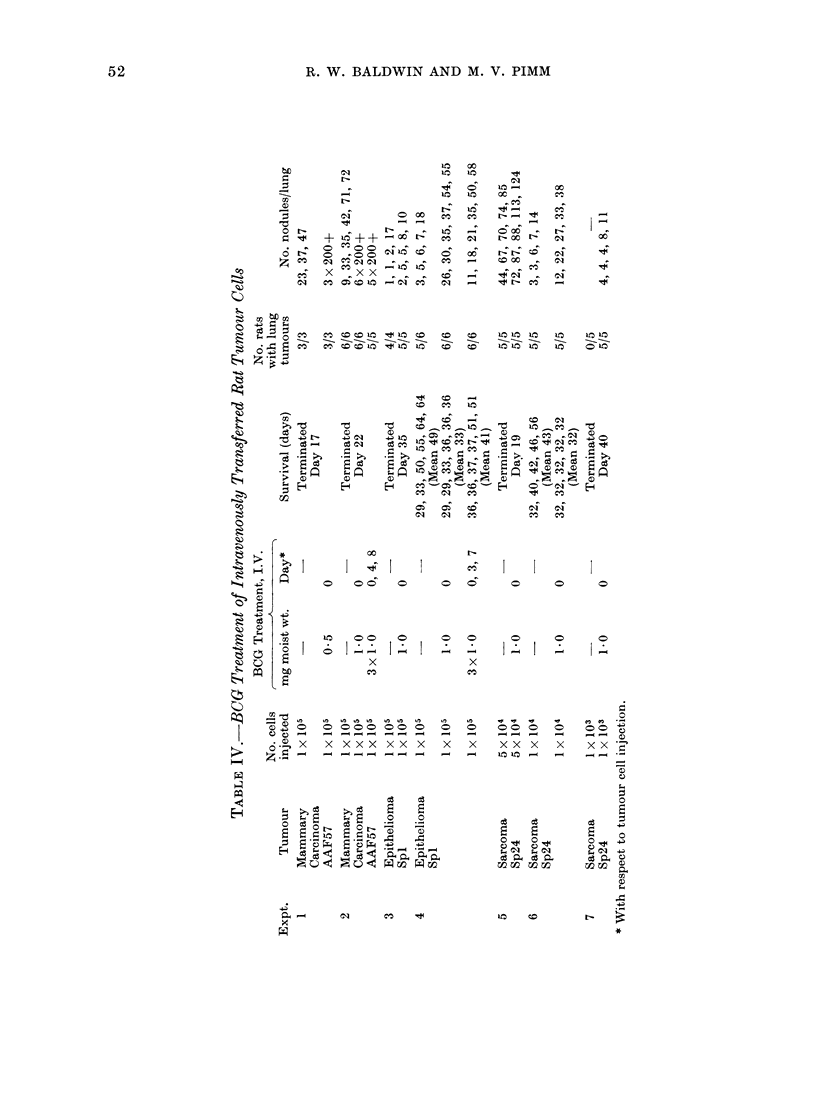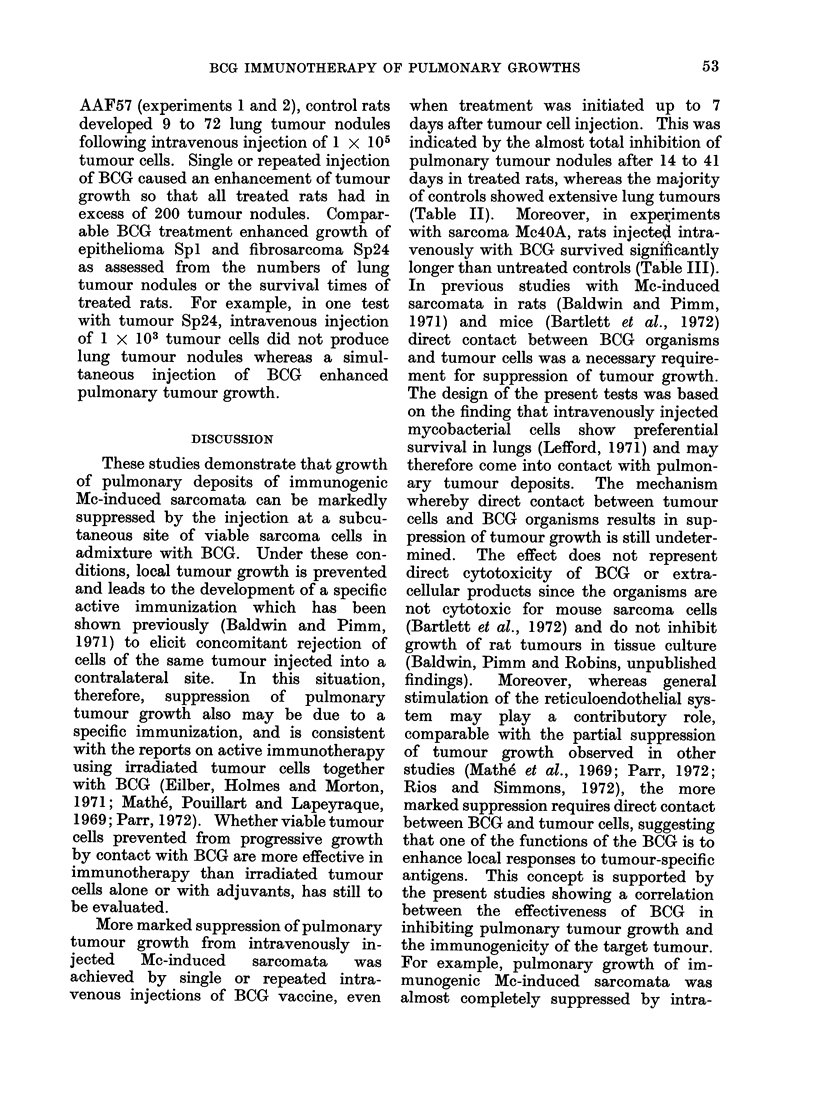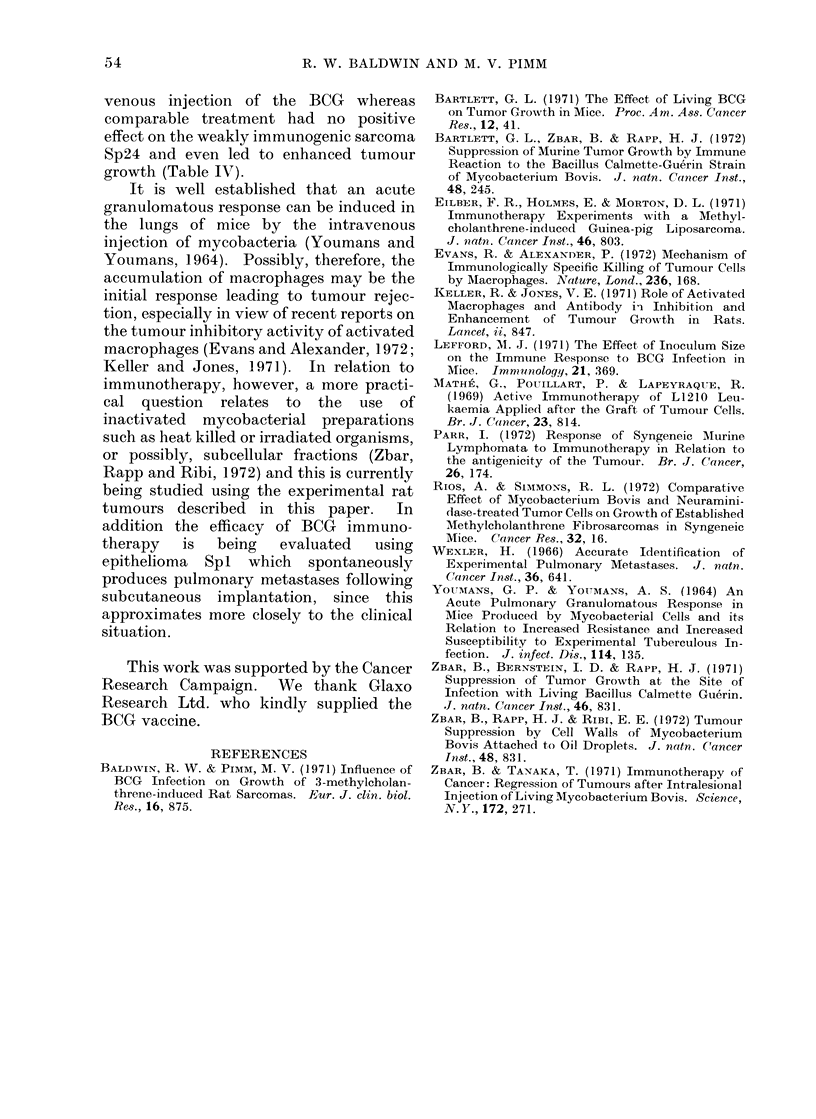# BCG Immunotherapy of Pulmonary Growths from Intravenously Transferred Rat Tumour Cells

**DOI:** 10.1038/bjc.1973.6

**Published:** 1973-01

**Authors:** R. W. Baldwin, M. V. Pimm

## Abstract

Pulmonary growths produced by intravenous injection of methycholanthrene-induced sarcoma cells were controlled or suppressed by specific immunostimulation with BCG-sarcoma cell inocula at subcutaneous sites or by intravenous treatment with BCG alone. The response was dependent upon the immunogenicity of the treated tumour so that in comparison, intravenous injection of BCG enhanced rather than suppressed pulmonary growths of transferred cells of several weakly immunogenic tumours including a spontaneously arising sarcoma and an epithelioma as well as a carcinogen-induced mammary carcinoma.


*Br. J. Cancer* (1973) **27**, 48

### BCG IMMUNOTHERAPY OF PULMONARY GROWTHS FROM INTRAVENOUSLY TRANSFERRED RAT TUMOUR CELLS

R. W. BALDWIN AND M. V. PIMM

*From the Cancer Research Campaign Laboratories, University of Nottingham, Nottingham NG7 2RD*

Received 26 September 1972. Accepted 9 October 1972

**Summary.—Pulmonary growths produced by intravenous injection of methycholanthrene-induced sarcoma cells were controlled or suppressed by specific immunostimulation with BCG-sarcoma cell inocula at subcutaneous sites or by intravenous treatment with BCG alone. The response was dependent upon the immunogenicity of the treated tumour so that in comparison, intravenous injection of BCG enhanced rather than suppressed pulmonary growths of transferred cells of several weakly immunogenic tumours including a spontaneously arising sarcoma and an epithelioma as well as a carcinogen-induced mammary carcinoma.**

STUDIES with guinea-pig hepatomata (Zbar, Bernstein and Rapp, 1971) and 3-methylcholanthrene (Mc)-induced sarcomata in rats (Baldwin and Pimm, 1971) and mice (Bartlett, 1971; Bartlett, Zbar and Rapp, 1972) have established that subcutaneous or intradermal implantation of tumour cells in admixture with viable Bacillus Calmette Guérin (BCG) organisms prevents their growth in genetically compatible hosts. Furthermore, deliberate infection of established local tumours may also lead to their suppression (Baldwin and Pimm, 1971; Zbar and Tanaka, 1971), and, with guinea-pig hepatomata, to the inhibition of lymph node metastasis (Zbar and Tanaka, 1971).

The mechanism of this tumour inhibition is still not understood, although factors other than general immunostimulation are involved, since intimate contact between BCG and tumour cells is necessary to suppress tumour growth (Baldwin and Pimm, 1971; Zbar *et al.*, 1971; Bartlett *et al.*, 1972). Nevertheless, specific responses to tumour-associated rejection antigens may be evoked, since rejection of cells of rat sarcomata (Baldwin and Pimm, 1971) and guinea-pig hepatomata (Zbar *et al.*, 1971) in admixture with BCG provides protection against challenge with cells of the same tumour, but not other antigenically distinct tumours.

The objective of the present investigation was to explore immunotherapeutic methods with BCG for the treatment of pulmonary deposits of tumour, produced by intravenous transfer of tumour cells. Whilst it is recognized that this mode of producing tumour growth in the lungs may differ from that occurring during spontaneous pulmonary metastasis, it has allowed a comparison of the effectiveness of BCG in the treatment of disseminated rat tumour cells of different histological types, with defined immunogenicities, ranging from highly immunogenic Mc-induced sarcomata, to weakly or non-immunogenic chemically-induced or spontaneously arising tumours.

### MATERIALS AND METHODS

### *Tumours*

The tumours used in these studies were induced with chemical carcinogens or arose without deliberate induction in rats of an inbred Wistar strain. Each tumour was carried by subcutaneous transplantation in syngeneic rats of the same sex as the primary donor. The following tumours were used:

(i) *Sarcomata Mc7, Mc40A and Mc52A*—induced by subcutaneous injection of 3-

methylcholanthrene. These tumours were highly antigenic, animals immunized by excision of subcutaneous growths subsequently rejecting challenge with whole tumour grafts.

(ii) *Mammary carcinoma AAF57*—induced by oral administration of N-hydroxy-2-acetylaminofluorene. This tumour lacked significant antigenicity, since excision of subcutaneous grafts did not elicit resistance against a subsequent challenge with $5 \times 10^4$ viable cells.

(iii) *Sarcoma Sp24*—a fibrosarcoma, arising spontaneously and weakly immunogenic, immunized rats rejecting only $10^3$ viable cells.

(iv) *Epithelioma Sp1*—arose spontaneously and exhibited low antigenicity, no more than $5 \times 10^4$ cells being rejected by immunized rats.

Single cell suspensions of tumours were prepared by digestion of finely minced tissue with 0·25% trypsin in Hank's balanced salt solution and resuspension in medium 199. Pulmonary growth of tumours was produced by intravenous injection of single cell suspensions into a lateral tail vein.

*Bacillus Calmette Guérin (BCG)*

Freeze-dried BCG vaccine (Percutaneous) was supplied by Glaxo Laboratories Ltd., Greenford, Middlesex, England. The vaccine was reconstituted in water to 10 mg moist weight of organisms/ml.

*Methods of treatment*

Active immunotherapy of intravenously transferred tumour cells was given by single or repeated subcutaneous injections of tumour cells in admixture with viable BCG, growth of these inocula being completely prevented.

Adjuvant immunotherapy by systemic BCG infection was effected by single or repeated intravenous injections of BCG (0·1 mg to 1·0 mg moist weight). When this treatment was given at the same time as intravenous tumour cell injection, BCG and tumour cells were injected in admixture.

*Assessment of pulmonary tumour growth*

Pulmonary growth of intravenously transferred tumour cells was demonstrated by perfusion of lungs with diluted Indian ink, followed by fixation in Fekete's solution (Wexler, 1966), and the number of macroscopic nodules on the lung surface counted.

In those experiments where the influence of BCG treatment on survival after intravenous injection of tumour cells was examined, animals were killed individually when they became distressed due to pulmonary tumour growth. Survival times were calculated with respect to the day of tumour cell injection.

### RESULTS

### *Immunotherapy of pulmonary deposits of 3-methylcholanthrene-induced sarcomata*

*Active immunotherapy.*—In the initial series of experiments, cells of Mc-induced sarcomata were injected intravenously and their pulmonary growth treated by active immunotherapy. This was given by the

TABLE I.—*Active Immunotherapy of Intravenously Transferred Rat Sarcoma Cells*

| Expt. | Sarcoma | No. cells injected | Treatment: Day* | Treatment: Inoculum† | Expt. terminated (day*) | No. rats with lung tumours | No. nodules/lung |
|---|---|---|---|---|---|---|---|
| 1 | Mc40A | $5 \times 10^6$ | 0 | 1·5 mg BCG+ $5 \times 10^6$ Mc40A cells | 14 | 1/5 | 1 × 200+ |
| | | $5 \times 10^6$ | — | — | 14 | 5/5 | 5 × 200+ |
| 2 | Mc40A | $5 \times 10^5$ | 0 | 1·0 mg BCG+ $3 \times 10^6$ Mc40A cells | 19 | 1/4 | 1 × 70 |
| | | $5 \times 10^5$ | — | — | 19 | 4/5 | 4 × 200+ |
| 3 | Mc52A | $1 \times 10^6$ | 0 | 1·0 mg BCG+ $1 \times 10^6$ Mc52A cells | 21 | 2/4 | 48, 200+ |
| | | $1 \times 10^6$ | 10 | 1·0 mg BCG+ $1 \times 10^6$ Mc52A cells | 21 | 2/4 | 75, 200+ |
| | | $1 \times 10^6$ | — | — | 21 | 4/4 | 3, 3 × 200+ |
| 4 | Mc52A | $2 \times 10^6$ | 0 | 1·0 mg BCG+ $2 \times 10^6$ Mc52A cells | 41 | 0/5 | — |
| | | $2 \times 10^6$ | — | — | 41 | 3/4 | 2, 3, 20 |

* With respect to tumour cell injection.
† Viable sarcoma cells in admixture with BCG injected subcutaneously.

subcutaneous injection of viable cells of the same tumour (1 to 5 $\times$ $10^6$ cells) in admixture with BCG (1·0 to 1·5 mg moist weight), since it has been established that such inocula do not develop and produce an effective immune response capable of suppressing subcutaneous sarcoma growth at a distant site (Baldwin and Pimm, 1971). The results, summarized in Table I, clearly indicate that when sarcoma cells and BCG were inoculated subcutaneously at the same time as tumour cells were given intravenously, pulmonary tumour growth was inhibited.

In experiment 1, 5 $\times$ $10^6$ sarcoma Mc40A cells were injected intravenously, and the rats simultaneously received a subcutaneous injection of 5 $\times$ $10^6$ viable Mc40A cells mixed with 1·5 mg moist weight of BCG. The experiment was terminated 14 days later, since control rats showed respiratory distress, all having multiple (200+) tumour nodules in the lungs. In contrast, only one of 5 animals receiving immunotherapy had pulmonary deposits of tumour.

In 3 further experiments with sarcomata Mc40A and Mc52A, active immunotherapy prevented pulmonary tumour development in the majority (12/17) of animals. Furthermore, with sarcoma Mc52A partial suppression was obtained even when treatment was delayed until 10 days after intravenous injection of sarcoma cells (experiment 3).

*Adjuvant immunotherapy.*—Previous studies (Baldwin and Pimm, 1971) showed that direct contact between BCG organisms and sarcoma cells was necessary to inhibit local tumour growth. Tests were therefore carried out to evaluate whether pulmonary growth of intravenously transferred sarcoma cells could be inhibited by systemic treatment with BCG, itself administered intravenously. In these tests sarcoma cells (5 $\times$ $10^5$ to 5 $\times$ $10^6$) were inoculated intravenously in admixture with BCG (0·1 mg or 1·0 mg moist weight), or BCG (1·0 mg moist weight) was injected separately 5 to 7 days after tumour cell injection (Table II). Almost all (30/32) control rats developed pulmonary tumour nodules, so that the experiments had to be terminated 14 to 41 days after tumour cell injections. In contrast only 3/56 animals receiving an intravenous injection of BCG, either together with the tumour cells, or 5 to 7

TABLE II.—*BCG Adjuvant Treatment of Intravenously Transferred Rat Sarcoma Cells*

| | | | BCG treatment | | | | |
|---|---|---|---|---|---|---|---|
| Expt. | Sarcoma | No. cells injected | Day* | Dose (mg moist weight) | Expt. terminated (day*) | No. rats with lung tumours | No. nodules/lung |
| 1 | Mc40A | $5 \times 10^6$ | — | — | 14 | 5/5 | $5 \times 200+$ |
| | | | 0 | 1·0 | 14 | 0/5 | — |
| 2 | Mc40A | $5 \times 10^5$ | — | — | 19 | 4/5 | $4 \times 200+$ |
| | | | 0 | 1·0 | 19 | 0/5 | — |
| | | | 5 | 1·0 | 19 | 0/5 | — |
| 3 | Mc40A | $5 \times 10^5$ | — | — | 21 | 5/5 | 40, 140, $3 \times 200+$ |
| | | | 0 | 1·0 | 21 | 0/5 | — |
| | | | 6 | 1·0 | 21 | 0/5 | — |
| 4 | Mc40A | $5 \times 10^5$ | — | — | 32 | 5/5 | 5, 31, 73, 100, 120 |
| | | | 0 | 1·0 | 32 | 3/5 | 2, 6, 13 |
| | | | 0 | 0·1 | 32 | 0/5 | — |
| 5 | Mc7 | $2 \times 10^6$ | — | — | 14 | 4/4 | 40, 140, $2 \times 200+$ |
| | | | 0 | 1·0 | 14 | 0/4 | — |
| | | | 7 | 1·0 | 14 | 0/4 | — |
| 6 | Mc52A | $1 \times 10^6$ | — | — | 21 | 4/4 | 3, $3 \times 200+$ |
| | | | 0 | 1·0 | 21 | 0/4 | — |
| | | | 6 | 1·0 | 21 | 0/5 | — |
| 7 | Mc52A | $2 \times 10^6$ | — | — | 41 | 3/4 | 2, 3, 20 |
| | | | 0 | 1·0 | 41 | 0/4 | — |

* With respect to tumour cell injection.

TABLE III.—*Immunotherapy of Intravenously Transferred Sarcoma Mc40A Cells**

| Expt. | Day† | Treatment Route | Treatment Inoculum | Survival (days) | No. rats with lung tumours | No. nodules/lung |
|---|---|---|---|---|---|---|
| 1 | 0, 3, 5, 7 | I.V. | 4 × 1 mg BCG | 49, 52, 52, 94, 129 (Mean 75) | 4/5 | 2, 6, 10, 30 |
| | 0, 5, 10 | S.C. | 3 × 1 mg BCG + 2 × $10^6$ Mc40A cells | 33, 43, 48, 54 (Mean 44) | 4/4 | 45, 72, 120, 200+ |
| | — | — | — | 25, 31, 32, 33 (Mean 30) | 4/4 | 116, 3 × 200+ |
| 2‡ | 0, 6, 10, 12 | I.V. | 4 × 1 mg BCG | 57, 4 × 100 (Mean 91) | 1/5 | 4 |
| | 6, 10, 12, 15 | I.V. | 4 × 1 mg BCG | 56, 57, 2 × 100 (Mean 78) | 0/4 | — |
| | 0 | I.V. | 1 × 1 mg BCG | 4 × 100 (Mean 100) | 2/4 | 1, 2 |
| | — | — | — | 21, 21, 22, 37 (Mean 25) | 4/4 | 130, 3 × 200+ |

* 5 × $10^5$ sarcoma Mc40A cells
† With respect to tumour cell injection.
‡ Experiment terminated at Day 100.

days later, had macroscopic tumour nodules in the lungs when the experiments were terminated.

In a further series of experiments with sarcoma Mc40A, an assessment was made of the effect of immunotherapy on the survival of rats following the intravenous injection of tumour cells (Table III). Both active immunotherapy and systemic treatment with BCG markedly prolonged survival following the intravenous injection of 5 × $10^5$ sarcoma cells.

In experiment 1, control animals had to be killed 25 to 33 days (mean 30 days) after intravenous tumour cell injection due to the development of multiple pulmonary deposits (116 to 200 nodules/lung). Animals receiving 3 subcutaneous injections of 2 × $10^6$ viable Mc40A cells in admixture with BCG (1·0 mg moist weight) at 5-day intervals survived for 33 to 54 days (mean 44 days) and had fewer lung tumour nodules (45 to 200 nodules). Four intravenous injections of BCG (1·0 mg moist weight) at 2 to 3 day intervals, starting at the time of intravenous tumour cell injection, produced a more marked prolongation of survival (49 to 129 days, mean 75 days) and a considerable reduction in the numbers of pulmonary tumour nodules (0 to 30 nodules).

In the second experiment, control animals survived for 21 to 37 days (mean 25 days) and developed multiple pulmonary tumour deposits (130 to 200 nodules/lung). A single injection of BCG at the same time as tumour cell injection, or repeated administration starting up to 6 days later, again significantly prolonged survival and markedly reduced the numbers of pulmonary tumour deposits. Thus only 3/13 treated rats developed tumour nodules in the lung (1 to 4 nodules/lung), the majority of animals remaining healthy and tumour free for 100 days, at which time the experiment was terminated.

*Immunotherapy of pulmonary deposits of other tumours*

In view of the inhibitory effect of the systemically administered BCG on the growth of intravenously transferred cells of highly immunogenic sarcomata, the effectiveness of this form of treatment was evaluated using tumours with lower immunogenicities. In these tests, tumour cells (1 × $10^3$ to 1 × $10^5$) were injected intravenously in admixture with BCG (0·5 mg to 1·0 mg moist weight) and in some cases, rats received 2 further BCG injections at 3 to 4 day intervals (Table IV).

With all 3 tumours tested, pulmonary tumour growth was consistently enhanced rather than inhibited by BCG treatment. Thus in 2 tests with mammary carcinoma

TABLE IV.—*BCG Treatment of Intravenously Transferred Rat Tumour Cells*

| Expt. | Tumour | No. cells injected | BCG Treatment, I.V. mg moist wt. | BCG Treatment, I.V. Day* | Survival (days) | No. rats with lung tumours | No. nodules/lung |
|---|---|---|---|---|---|---|---|
| 1 | Mammary Carcinoma | $1 \times 10^5$ | — | — | Terminated Day 17 | 3/3 | 23, 37, 47 |
| | AAF57 | $1 \times 10^5$ | 0·5 | 0 | | 3/3 | $3 \times 200+$ |
| 2 | Mammary | $1 \times 10^5$ | — | — | Terminated | 6/6 | 9, 33, 35, 42, 71, 72 |
| | Carcinoma | $1 \times 10^5$ | 1·0 | 0 | Day 22 | 6/6 | $6 \times 200+$ |
| | AAF57 | $1 \times 10^5$ | $3 \times 1·0$ | 0, 4, 8 | | 5/5 | $5 \times 200+$ |
| 3 | Epithelioma | $1 \times 10^5$ | — | — | Terminated | 4/4 | 1, 1, 2, 17 |
| | Sp1 | $1 \times 10^5$ | 1·0 | 0 | Day 35 | 5/5 | 2, 5, 5, 8, 10 |
| 4 | Epithelioma Sp1 | $1 \times 10^5$ | — | — | 29, 33, 50, 55, 64, 64 (Mean 49) | 5/6 | 3, 5, 6, 7, 18 |
| | | $1 \times 10^5$ | 1·0 | 0 | 29, 29, 33, 36, 36, 36 (Mean 33) | 6/6 | 26, 30, 35, 37, 54, 55 |
| | | $1 \times 10^5$ | $3 \times 1·0$ | 0, 3, 7 | 36, 36, 37, 37, 51, 51 (Mean 41) | 6/6 | 11, 18, 21, 35, 50, 58 |
| 5 | Sarcoma | $5 \times 10^4$ | — | — | Terminated | 5/5 | 44, 67, 70, 74, 85 |
| | Sp24 | $5 \times 10^4$ | 1·0 | 0 | Day 19 | 5/5 | 72, 87, 88, 113, 124 |
| 6 | Sarcoma Sp24 | $1 \times 10^4$ | — | — | 32, 40, 42, 46, 56 (Mean 43) | 5/5 | 3, 3, 6, 7, 14 |
| | | $1 \times 10^4$ | 1·0 | 0 | 32, 32, 32, 32, 32 (Mean 32) | 5/5 | 12, 22, 27, 33, 38 |
| 7 | Sarcoma | $1 \times 10^3$ | — | — | Terminated | 0/5 | — |
| | Sp24 | $1 \times 10^3$ | 1·0 | 0 | Day 40 | 5/5 | 4, 4, 4, 8, 11 |

* With respect to tumour cell injection.

AAF57 (experiments 1 and 2), control rats developed 9 to 72 lung tumour nodules following intravenous injection of $1 \times 10^5$ tumour cells. Single or repeated injection of BCG caused an enhancement of tumour growth so that all treated rats had in excess of 200 tumour nodules. Comparable BCG treatment enhanced growth of epithelioma Sp1 and fibrosarcoma Sp24 as assessed from the numbers of lung tumour nodules or the survival times of treated rats. For example, in one test with tumour Sp24, intravenous injection of $1 \times 10^3$ tumour cells did not produce lung tumour nodules whereas a simultaneous injection of BCG enhanced pulmonary tumour growth.

### DISCUSSION

These studies demonstrate that growth of pulmonary deposits of immunogenic Mc-induced sarcomata can be markedly suppressed by the injection at a subcutaneous site of viable sarcoma cells in admixture with BCG. Under these conditions, local tumour growth is prevented and leads to the development of a specific active immunization which has been shown previously (Baldwin and Pimm, 1971) to elicit concomitant rejection of cells of the same tumour injected into a contralateral site. In this situation, therefore, suppression of pulmonary tumour growth also may be due to a specific immunization, and is consistent with the reports on active immunotherapy using irradiated tumour cells together with BCG (Eilber, Holmes and Morton, 1971; Mathé, Pouillart and Lapeyraque, 1969; Parr, 1972). Whether viable tumour cells prevented from progressive growth by contact with BCG are more effective in immunotherapy than irradiated tumour cells alone or with adjuvants, has still to be evaluated.

More marked suppression of pulmonary tumour growth from intravenously injected Mc-induced sarcomata was achieved by single or repeated intravenous injections of BCG vaccine, even when treatment was initiated up to 7 days after tumour cell injection. This was indicated by the almost total inhibition of pulmonary tumour nodules after 14 to 41 days in treated rats, whereas the majority of controls showed extensive lung tumours (Table II). Moreover, in experiments with sarcoma Mc40A, rats injected intravenously with BCG survived significantly longer than untreated controls (Table III). In previous studies with Mc-induced sarcomata in rats (Baldwin and Pimm, 1971) and mice (Bartlett *et al.*, 1972) direct contact between BCG organisms and tumour cells was a necessary requirement for suppression of tumour growth. The design of the present tests was based on the finding that intravenously injected mycobacterial cells show preferential survival in lungs (Lefford, 1971) and may therefore come into contact with pulmonary tumour deposits. The mechanism whereby direct contact between tumour cells and BCG organisms results in suppression of tumour growth is still undetermined. The effect does not represent direct cytotoxicity of BCG or extracellular products since the organisms are not cytotoxic for mouse sarcoma cells (Bartlett *et al.*, 1972) and do not inhibit growth of rat tumours in tissue culture (Baldwin, Pimm and Robins, unpublished findings). Moreover, whereas general stimulation of the reticuloendothelial system may play a contributory role, comparable with the partial suppression of tumour growth observed in other studies (Mathé *et al.*, 1969; Parr, 1972; Rios and Simmons, 1972), the more marked suppression requires direct contact between BCG and tumour cells, suggesting that one of the functions of the BCG is to enhance local responses to tumour-specific antigens. This concept is supported by the present studies showing a correlation between the effectiveness of BCG in inhibiting pulmonary tumour growth and the immunogenicity of the target tumour. For example, pulmonary growth of immunogenic Mc-induced sarcomata was almost completely suppressed by intravenous injection of the BCG whereas comparable treatment had no positive effect on the weakly immunogenic sarcoma Sp24 and even led to enhanced tumour growth (Table IV).

It is well established that an acute granulomatous response can be induced in the lungs of mice by the intravenous injection of mycobacteria (Youmans and Youmans, 1964). Possibly, therefore, the accumulation of macrophages may be the initial response leading to tumour rejection, especially in view of recent reports on the tumour inhibitory activity of activated macrophages (Evans and Alexander, 1972; Keller and Jones, 1971). In relation to immunotherapy, however, a more practical question relates to the use of inactivated mycobacterial preparations such as heat killed or irradiated organisms, or possibly, subcellular fractions (Zbar, Rapp and Ribi, 1972) and this is currently being studied using the experimental rat tumours described in this paper. In addition the efficacy of BCG immunotherapy is being evaluated using epithelioma Sp1 which spontaneously produces pulmonary metastases following subcutaneous implantation, since this approximates more closely to the clinical situation.

This work was supported by the Cancer Research Campaign. We thank Glaxo Research Ltd. who kindly supplied the BCG vaccine.

### REFERENCES

BALDWIN, R. W. & PIMM, M. V. (1971) Influence of BCG Infection on Growth of 3-methylcholanthrene-induced Rat Sarcomas. *Eur. J. clin. biol. Res.*, **16**, 875.

BARTLETT, G. L. (1971) The Effect of Living BCG on Tumor Growth in Mice. *Proc. Am. Ass. Cancer Res.*, **12**, 41.

BARTLETT, G. L., ZBAR, B. & RAPP, H. J. (1972) Suppression of Murine Tumor Growth by Immune Reaction to the Bacillus Calmette-Guérin Strain of Mycobacterium Bovis. *J. natn. Cancer Inst.*, **48**, 245.

EILBER, F. R., HOLMES, E. & MORTON, D. L. (1971) Immunotherapy Experiments with a Methylcholanthrene-induced Guinea-pig Liposarcoma. *J. natn. Cancer Inst.*, **46**, 803.

EVANS, R. & ALEXANDER, P. (1972) Mechanism of Immunologically Specific Killing of Tumour Cells by Macrophages. *Nature, Lond.*, **236**, 168.

KELLER, R. & JONES, V. E. (1971) Role of Activated Macrophages and Antibody in Inhibition and Enhancement of Tumour Growth in Rats. *Lancet*, *ii*, 847.

LEFFORD, M. J. (1971) The Effect of Inoculum Size on the Immune Response to BCG Infection in Mice. *Immunology*, **21**, 369.

MATHÉ, G., POUILLART, P. & LAPEYRAQUE, R. (1969) Active Immunotherapy of L1210 Leukaemia Applied after the Graft of Tumour Cells. *Br. J. Cancer*, **23**, 814.

PARR, I. (1972) Response of Syngeneic Murine Lymphomata to Immunotherapy in Relation to the antigenicity of the Tumour. *Br. J. Cancer*, **26**, 174.

RIOS, A. & SIMMONS, R. L. (1972) Comparative Effect of Mycobacterium Bovis and Neuraminidase-treated Tumor Cells on Growth of Established Methylcholanthrene Fibrosarcomas in Syngeneic Mice. *Cancer Res.*, **32**, 16.

WEXLER, H. (1966) Accurate Identification of Experimental Pulmonary Metastases. *J. natn. Cancer Inst.*, **36**, 641.

YOUMANS, G. P. & YOUMANS, A. S. (1964) An Acute Pulmonary Granulomatous Response in Mice Produced by Mycobacterial Cells and its Relation to Increased Resistance and Increased Susceptibility to Experimental Tuberculous Infection. *J. infect. Dis.*, **114**, 135.

ZBAR, B., BERNSTEIN, I. D. & RAPP, H. J. (1971) Suppression of Tumor Growth at the Site of Infection with Living Bacillus Calmette Guérin. *J. natn. Cancer Inst.*, **46**, 831.

ZBAR, B., RAPP, H. J. & RIBI, E. E. (1972) Tumour Suppression by Cell Walls of Mycobacterium Bovis Attached to Oil Droplets. *J. natn. Cancer Inst.*, **48**, 831.

ZBAR, B. & TANAKA, T. (1971) Immunotherapy of Cancer: Regression of Tumours after Intralesional Injection of Living Mycobacterium Bovis. *Science, N.Y.*, **172**, 271.